# Rational attenuation of canine distemper virus (CDV) to develop a morbillivirus animal model that mimics measles in humans

**DOI:** 10.1128/jvi.01850-23

**Published:** 2024-02-28

**Authors:** Katharina S. Schmitz, Linda J. Rennick, Natasha L. Tilston-Lunel, Anouskha D. Comvalius, Brigitta M. Laksono, Daryl Geers, Peter van Run, Rory D. de Vries, Rik L. de Swart, W. Paul Duprex

**Affiliations:** 1 Department of Viroscience, Erasmus MC, Rotterdam, the Netherlands; 2 Center for Vaccine Research, University of Pittsburgh School of Medicine, Pittsburgh, Pennsylvania, USA; University of Kentucky College of Medicine, Lexington, Kentucky, USA

**Keywords:** measles, canine distemper, ferret, model, attenuation

## Abstract

Morbilliviruses are members of the family Paramyxoviridae and are known for their ability to cause systemic disease in a variety of mammalian hosts. The prototypic morbillivirus, measles virus (MeV), infects humans and still causes morbidity and mortality in unvaccinated children and young adults. Experimental infection studies in non-human primates have contributed to the understanding of measles pathogenesis. However, ethical restrictions call for the development of new animal models. Canine distemper virus (CDV) infects a wide range of animals, including ferrets, and its pathogenesis shares many features with measles. However, wild-type CDV infection is almost always lethal, while MeV infection is usually self-limiting. Here, we made five recombinant CDVs, predicted to be attenuated, and compared their pathogenesis to the non-attenuated recombinant CDV in a ferret model. Three viruses were insufficiently attenuated based on clinical signs, fatality, and systemic infection, while one virus was too attenuated. The last candidate virus caused a self-limiting infection associated with transient viremia and viral dissemination to all lymphoid tissues, was shed transiently from the upper respiratory tract, and did not result in acute neurological signs. Additionally, an in-depth phenotyping of the infected white blood cells showed lower infection percentages in all lymphocyte subsets when compared to the non-attenuated CDV. In conclusion, infection models using this candidate virus mimic measles and can be used to study pathogenesis-related questions and to test interventions for morbilliviruses in a natural host species.

**IMPORTANCE:**

Morbilliviruses are transmitted via the respiratory route but cause systemic disease. The viruses use two cellular receptors to infect myeloid, lymphoid, and epithelial cells. Measles virus (MeV) remains an important cause of morbidity and mortality in humans, requiring animal models to study pathogenesis or intervention strategies. Experimental MeV infections in non-human primates are restricted by ethical and practical constraints, and animal morbillivirus infections in natural host species have been considered as alternatives. Inoculation of ferrets with wild-type canine distemper virus (CDV) has been used for this purpose, but in most cases, the virus overwhelms the immune system and causes highly lethal disease. Introduction of an additional transcription unit and an additional attenuating point mutation in the polymerase yielded a candidate virus that caused self-limiting disease with transient viremia and virus shedding. This rationally attenuated CDV strain can be used for experimental morbillivirus infections in ferrets that reflect measles in humans.

## INTRODUCTION

Measles virus (MeV) is an airborne morbillivirus in the family Paramyxoviridae (1) and causes an acute disease characterized by fever and rash. The introduction of live-attenuated measles vaccines in the 1960s significantly reduced measles morbidity and is estimated to have averted more than 55 million deaths between 2000 and 2021 globally (2). After a global measles surge from 2017 to 2019, case numbers dropped again in 2020 and 2021 as a result of non-pharmaceutical COVID-19 interventions. However, the pandemic also resulted in reduced measles vaccination coverage, leaving more than 25 million children at risk for measles (2).

Initial MeV infection and replication are largely restricted to cell types that express the cellular entry receptor human signaling lymphocyte activation molecule F1 (hSLAMF1 or CD150). MeV infection is initiated in myeloid cells like dendritic cells (DCs) and alveolar macrophages (3, 4). Systemic dissemination is facilitated by circulating CD150-expressing B- and T-lymphocytes, whose depletion results in transient lymphopenia and immune suppression (5, 6). This causes increased susceptibility to secondary infections, which are responsible for the majority of measles-associated morbidity and mortality. Peripheral blood lymphocyte cell counts are usually restored within 2–3 weeks post-infection, but the immune suppression can last up to 2–3 years after measles (7). The preferential infection and depletion of CD150-expressing memory lymphocytes are the underlying cause of “immune amnesia”, a status in which lymphocyte numbers are restored, but the pre-measles immune repertoire is impaired (5, 6, 8–10). Ultimately, infiltration of MeV-infected immune cells into the respiratory submucosa results in infection of epithelial cells via nectin-4, a second cellular receptor that is part of the adherens junctions and, in differentiated epithelial cells, can only be reached from the basolateral side (11–13).

Unraveling the mechanisms underlying measles immune suppression was largely based on studies in experimentally infected macaques; the use of non-human primates (NHPs) in measles research has greatly facilitated our understanding of measles pathogenesis (14). However, NHP studies are becoming ethically and practically more challenging, resulting in a need for new animal models. Most rodents are not susceptible or permissive to wild-type (WT) MeV. Transgenic mice that overexpress the human MeV receptors are used as animal models (15, 16) but poorly recapitulate measles pathogenesis. Alternatively, animal morbilliviruses can be studied in natural hosts as a surrogate model for measles. Canine distemper virus (CDV) infects a broad host range of carnivores and omnivores, including dogs, ferrets, and raccoons (17, 18). CDV infection of ferrets has often been used as a surrogate model (19). CDV replication in ferrets largely resembles the natural course of MeV infection in primates (20). Importantly, while measles is usually not fatal in NHPs, WT CDV infection of ferrets can lead to high case fatality rates. Experimental infection of ferrets with a rationally attenuated CDV could be used as an improved animal model of disease for measles. This model could be used to address open questions such as the impact of measles on acquired immunity by vaccines and viral infections and, hence, inform if re-vaccination with typical childhood vaccines post measles should be recommended. Moreover, this model could also be used to study new strategies for measles prevention or treatment.

Several approaches have been used to attenuate morbilliviruses: (i) Morbillivirus replication is characterized by a transcriptional gradient, leading to more expression of the first encoded gene (nucleoprotein, N) than the gene encoded last (large protein, L) in the viral genome (21). Adding an additional transcription unit (ATU) proximal to N (position 1) attenuated CDV (22). (ii) Deleting the C protein may decrease CDV pathogenicity as this protein, expressed from an alternative open reading frame of the P gene, has been shown to be critical for MeV virulence (23). (iii) The insertion of enhanced green fluorescent protein (EGFP) into L was used to attenuate rinderpest virus (RPV), a morbillivirus closely related to MeV, and a highly pathogenic CDV strain (24, 25). (iv) A single-point mutation in L (H589Y) attenuated CDV in ferrets, resulting in the survival of two-thirds of animals, while all control animals succumbed to the infection (26). (v) Modifications of CDV hemagglutinin (H) rendered it unable to bind CD150 or nectin-4 (receptor-blind viruses) resulting in attenuated phenotypes (27, 28).

We set out to develop a rationally attenuated CDV that could be used for experimental infections of ferrets, resulting in an animal model of disease that mimics measles in humans. Before embarking on these studies, we defined a number of desired characteristics of the virus to resemble known properties of MeV as observed in humans and NHPs, based on previously published data (6–8, 27, 29–32): (i) CDV should use CD150 and nectin-4, and (ii) the infection should be self-limiting, i.e., non-lethal. CDV should cause (iii) transient lymphopenia, (iv) transient viremia, and should (v) result in spread to lymphoid tissues. Moreover, CDV should (vi) be transiently shed from the upper respiratory tract but (vii) not cause clinical signs that reflect acute central nervous system (CNS) infection.

A recombinant CDV (rCDV) containing an ATU encoding Venus fluorescent protein at position 6 (proximal to L) and based on a full-genome sequence obtained from a naturally infected raccoon in Rhode Island, USA, was recently generated [rCDV^RI^Venus(6)] (33). We studied the pathogenesis of this recombinant virus in raccoons (18) and ferrets (34). Here, we made five different recombinant CDVs based on this recombinant virus and compared attenuation of these viruses relative to infection with rCDV^RI^Venus(6) in ferrets. We evaluated clinical signs, case fatality, lymphopenia, viral shedding, viremia, and virus load in lymphoid tissues. Finally, we propose a recombinant CDV attenuated by insertion of an ATU at position 1 and an additional attenuating point mutation in L (H589Y) as a candidate virus to be used in a ferret model to mimic measles pathogenesis.

## RESULTS

### Clinical signs

**Fig 1:**
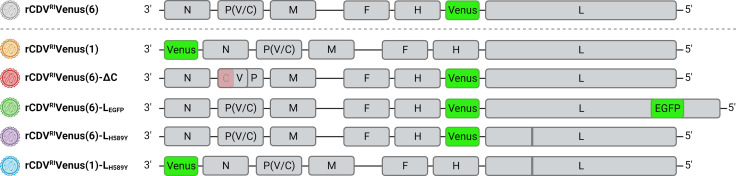
Schematic overview of rCDV constructs. Attempted rational attenuation of rCDV^RI^ by introducing an ATU (Venus) in position 1 (brown), or deleting the C protein (red), introducing EGFP into the L gene (green), or mutating position 589 (vertical line) of the L gene (purple) of rCDV^RI^Venus(6), or introducing Venus at position 1 and mutating position 589 of the L gene (blue). rCDV^RI^Venus(6), previously shown to be pathogenic in ferrets and raccoons, is shown in gray for comparison. The colors for the different rCDVs are consistent throughout the manuscript.

Five different rCDVs were made by inserting an ATU at position 1 [rCDV^RI^Venus(1)] (22), combining insertion of an ATU at position 6 or position 1 with an additional mutation in L [H589Y; rCDV^RI^Venus(6)-L_H589Y_ and rCDV^RI^Venus(1)-L_H589Y_] (26), combining insertion of an ATU at position 6 with insertion of EGFP into the polymerase [rCDV^RI^Venus(6)-L_EGFP_] (24, 25), or combining insertion of an ATU at position 6 with deletion of the C protein [rCDV^RI^Venus(6)-ΔC] (23) (Fig. 1). All recombinant viruses used the cellular receptors CD150 and nectin-4 (Fig. S1).

**Fig 2:**
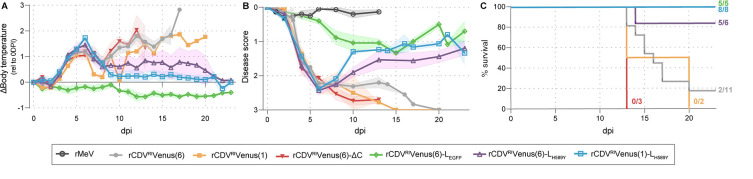
Clinical parameters of ferrets inoculated with different rCDVs. (**A**) Change in body temperature relative to day 0 for animals inoculated with different rCDVs (in degrees celcius). (**B**) The severity score, an average score of body temperature, body weight, lymphocyte count, virus isolation from throat swabs, and infection of lymphocytes for all rCDVs. The severity score of MeV-inoculated ferrets is shown as a negative control. Shaded bands depict the standard error of the mean (SEM). (**C**) Kaplan-Meier curve depicting the percentage survival of animals in each group.

Ferrets inoculated with rCDV^RI^Venus(6) presented with a fever from 3 dpi onward, which peaked at 6 dpi and stayed elevated until they reached their humane endpoints (Fig. 2A). This pattern was similar for animals inoculated with rCDV^RI^Venus(6)-ΔC and rCDV^RI^Venus(1). Five out of seven animals inoculated with rCDV^RI^Venus(6)-L_H589Y_ had a transient fever, while two animals presented with prolonged elevated body temperature until they reached their humane endpoint or the end of the study period was reached. All rCDV^RI^Venus(1)-L_H589Y_-inoculated ferrets experienced a transient fever peaking on day 6, whereas animals inoculated with rCDV^RI^Venus(6)-L_EGFP_ did not develop fever at all. Next, we determined the severity score (0–3), an average score of body temperature, body weight, lymphocyte counts, virus isolation from throat swabs, and infection of lymphocytes in blood (viremia) (Fig. 2B; Table S1). As MeV does not productively replicate in ferrets and therefore does not cause disease (34), we used MeV-inoculated ferrets, which had a stable severity score of 0, as controls. rCDV^RI^Venus(6)-L_EGFP_-inoculated ferrets had a peak score of 1. All other animals reached a severity score of >2 by day 6; animals inoculated with rCDV^RI^Venus(6)-L_H589Y_ and rCDV^RI^Venus(1)-L_H589Y_ partly recovered, while the severity score for rCDV^RI^Venus(6)-, rCDV^RI^Venus(6)-ΔC-, and rCDV^RI^Venus(1)-inoculated animals did not decrease. Ultimately, the severe phenotype of rCDV^RI^Venus(6)-ΔC and rCDV^RI^Venus(1) was confirmed as all animals reached their humane endpoints, while no ferrets succumbed to infection for the rCDV^RI^Venus(6)-L_EGFP_ variant or the rCDV^RI^Venus(1)-L_H589Y_ variant (Fig. 2C). One out of six ferrets inoculated with rCDV^RI^Venus(6)-L_H589Y_ reached its humane endpoints and was euthanized.

### CDV shedding

**Fig 3:**
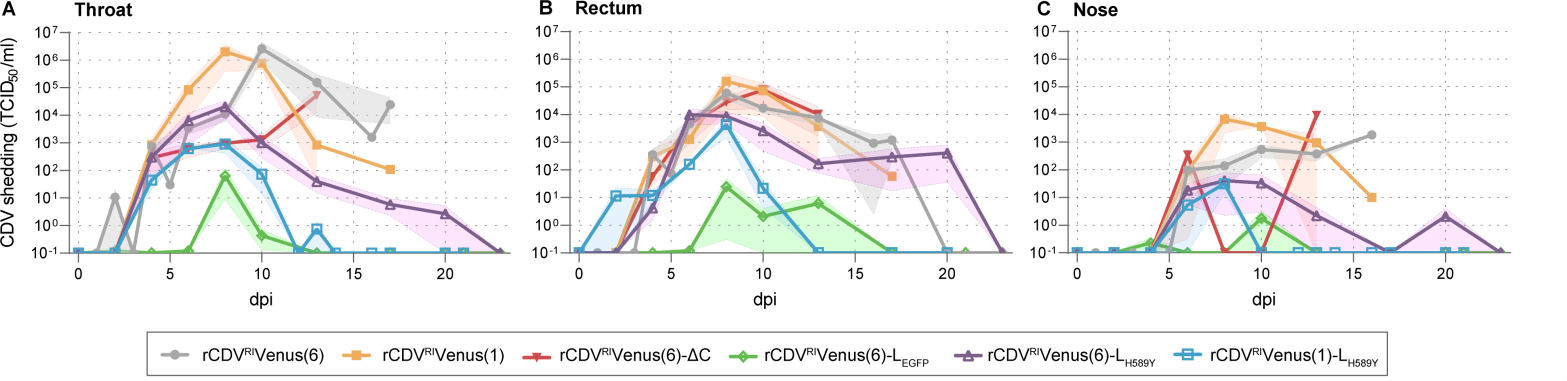
Shedding of rCDVs. Infectious rCDVs were isolated from (**A**) throat, (**B**) rectum, and (**C**) nose swabs on VDS cells. Titers were determined by endpoint titration and are expressed as TCID_50_/mL. Shaded bands depict the SEM.

We compared rCDV shedding patterns by obtaining throat, nose, and rectal swabs throughout the study period (Fig. 3). Generally, virus titers in the throat (Fig. 3A) were slightly higher than those obtained from rectal swabs (Fig. 3B). The lowest amount of virus was detected in the nose (Fig. 3C). rCDV^RI^Venus(6)-L_EGFP_ was shed for the shortest duration and to only low titers (<10^2^ TCID_50_/mL). In contrast, rCDV^RI^Venus(6), rCDV^RI^Venus(1), and rCDV^RI^Venus(6)-ΔC were all detected from 4 dpi onward until all animals reached their humane endpoints, and at their peak throat, nose, and rectal swab viral titers for these variants exceeded the titers observed for other rCDV variants. Shedding of both polymerase point mutation viruses was transient; however, shedding for rCDV^RI^Venus(6)-L_H589Y_ was of higher magnitude and longer duration when compared to rCDV^RI^Venus(1)-L_H589Y_.

### CDV viremia and lymphopenia

**Fig 4:**
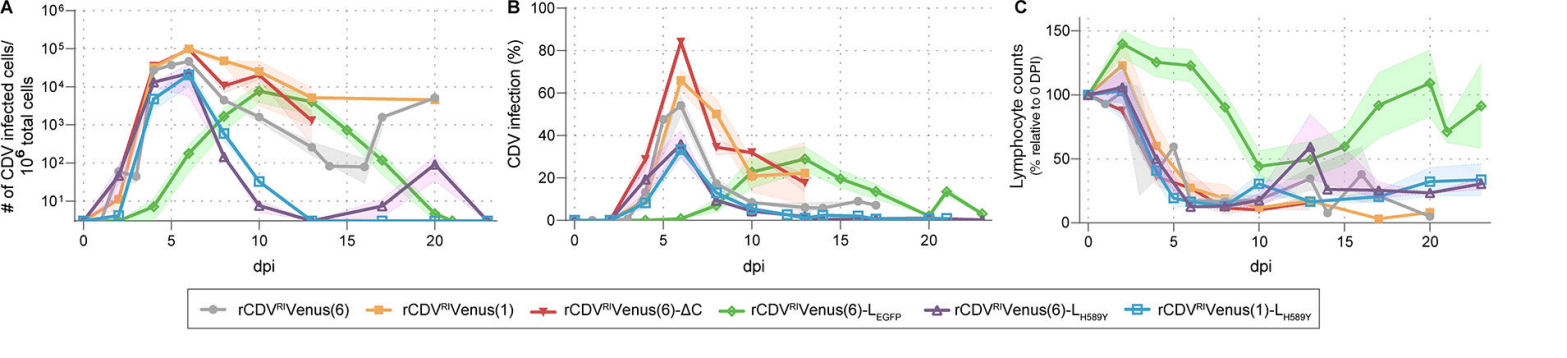
rCDV viremia and lymphopenia. Detection of rCDVs in peripheral blood via (**A**) virus isolation or (**B**) flow cytometry. (**A**) WBCs obtained from rCDV-inoculated ferrets were titrated on VDS cells, and viral loads were expressed as the number of infected cells per 10^6^ total cells. (**B**) Infection percentage of ferret lymphocytes measured by flow cytometry. The percentage of Venus^+^ cells in the singlet lymphocyte gate was determined. (**C**) Lymphocyte counts are shown relative to day 0 for animals inoculated with the different rCDVs. Shaded bands depict the SEM.

Next, we evaluated the infection of WBC and resulting lymphopenia in animals inoculated with the rCDVs (Fig. 4). To this end, we titrated WBC isolated from whole blood (Fig. 4A) and determined the infection percentage of lymphocytes by flow cytometry (Fig. 4B). The two variants that previously appeared insufficiently attenuated [rCDV^RI^Venus(6)-ΔC, rCDV^RI^Venus(1)], mimicked the pattern observed for rCDV^RI^Venus(6). Notably, at its peak at 6 dpi, lymphocyte infection percentages of rCDV^RI^Venus(6)-ΔC- and rCDV^RI^Venus(1)-inoculated animals exceeded those of the control group with >80% and >60%, respectively (Fig. 4B). Viremia of rCDV^RI^Venus(6)-L_EGFP_-inoculated animals appeared much later, and less virus could be isolated when compared to all other variants (Fig. 4A), which was also represented by the lower infection percentages (Fig. 4B). Viral titers from WBC and infection percentages of lymphocytes from ferrets inoculated with the polymerase point mutation viruses were similar. Virus was isolated from 2 dpi onward, and infectious virus loads peaked at 6 dpi (Fig. 4A). Peak infection percentages at 6 dpi were between 30% and 40% and declined afterward (Fig. 4B). With the exception of rCDV^RI^Venus(6)-L_EGFP_-inoculated animals, resulting changes in lymphocyte counts were similar for most animals (Fig. 4C). Lymphocyte counts dropped from 4 dpi onward, were lowest between 5 and 6 dpi, and stayed low for the entire study period. As an exception, lymphopenia of rCDV^RI^Venus(6)-L_EGFP_-inoculated ferrets was observed relatively late (from 8 dpi onward), and fully recovered by 20 dpi, additionally indicating mild disease in these ferrets as compared to animals inoculated with the other rCDVs.

Based on these findings, we identified rCDV^RI^Venus(1)-L_H589Y_ for in-depth characterization as a “MeV-like” virus; the other candidate viruses were either insufficiently [rCDV^RI^Venus(6)-ΔC, rCDV^RI^Venus(1)] or too attenuated (rCDV^RI^Venus(6)-L_EGFP_) and did not mimic measles pathogenesis (Fig. S2). Although rCDV^RI^Venus(6)-L_H589Y_ behaved similarly to rCDV^RI^Venus(1)-L_H589Y_ in our study, the higher case fatality rate (CFR) with CNS involvement (1/6) (Fig. 2C), the slightly higher overall severity score (Fig. 2B), and the prolonged shedding (Fig. 3) of the rCDV^RI^Venus(6)-L_H589Y_-inoculated ferrets made us choose rCDV^RI^Venus(1)-L_H589Y_ as our candidate-attenuated CDV.

### Phenotyping of CDV-infected white blood cells

**Fig 5:**
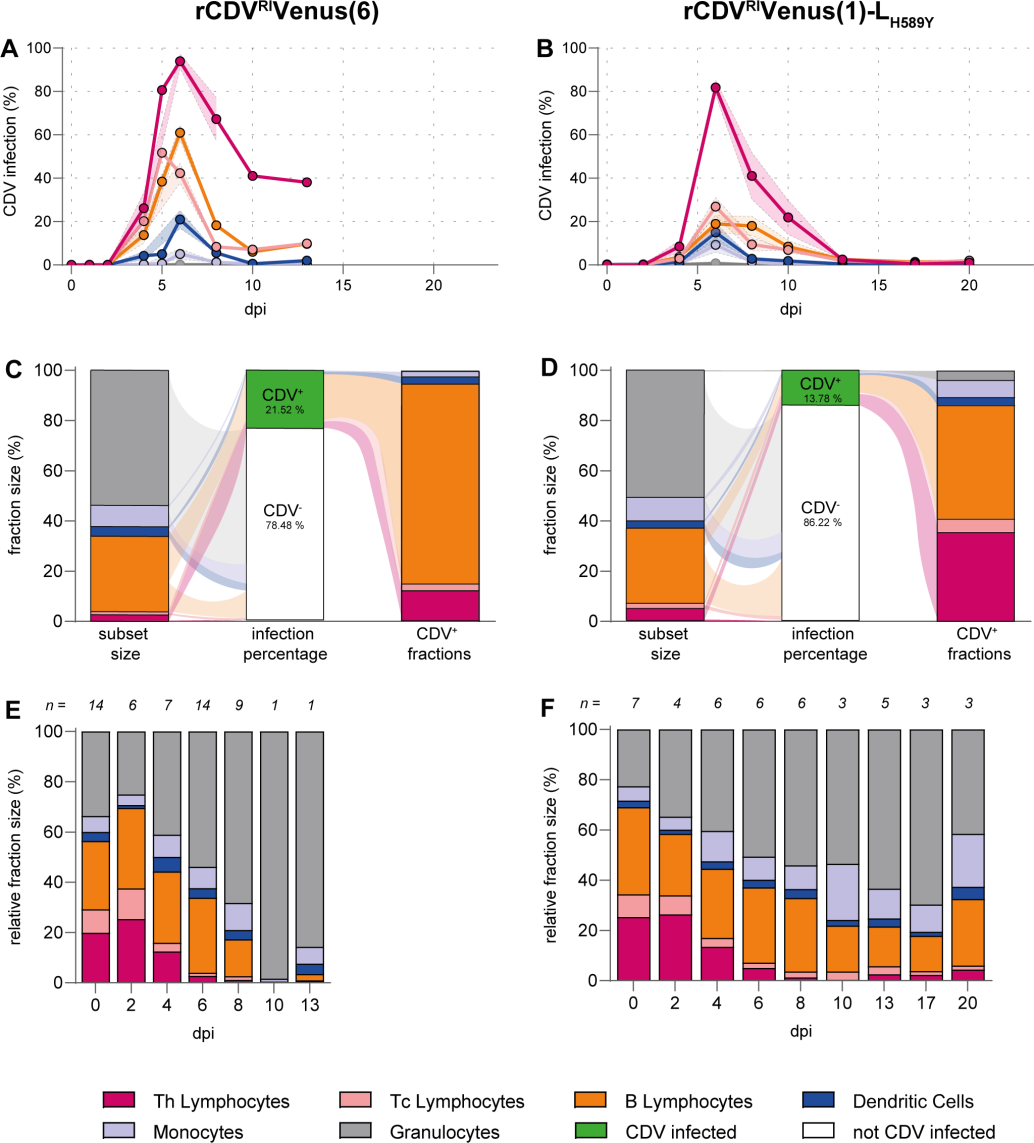
Phenotype of rCDV^RI^Venus(1)-L_H589Y_-infected WBC in comparison to rCDV^RI^Venus(6)-infected WBC. (**A and B**) Infection percentage of different cell types measured by flow cytometry. The percentage of Venus^+^ cells in the indicated population was determined for (**A**) rCDV^RI^Venus(6) and (**B**) rCDV^RI^Venus(1)-L_H589Y_. Per time point, all available samples were evaluated. Shaded bands indicate the SEM. (**C and D**) Phenotyping of rCDV-infected cells on 6 dpi via inverse gating of Venus^+^ cells within the singlet gate. The relative population sizes are shown as the average population sizes of (**C**) *n* = 14 rCDV^RI^Venus(6)-infected ferrets and (**D**) *n* = 6 rCDV^RI^Venus(1)-L_H589Y_-infected ferrets. (**E and F**) Subsets of WBC were identified in peripheral blood via flow cytometry at the indicated time points for (**E**) rCDV^RI^Venus(6) and (**F**) rCDV^RI^Venus(1)-L_H589Y_. The relative population size at each time point is shown as the average population size of all available samples per time point. The gating strategy can be found in Fig. S3.

Next, we characterized the phenotype of rCDV^RI^Venus(1)-L_H589Y_-infected cells and compared this to rCDV^RI^Venus(6)-infected cells. To this end, we identified WBCs as Th-lymphocytes, Tc-lymphocytes, B-lymphocytes, DCs, monocytes, and granulocytes using forward scatter (FSC), side scatter (SSC), and cell-surface markers (Fig. S3). For all subsets, we gated Venus^+^ cells to identify the target cells of rCDV^RI^Venus(1)-L_H589Y_. Similar to what was observed for rCDV^RI^Venus(6), the majority of infected cells were Th-lymphocytes (Fig. 5A and B). While on average, more than 90% of Th-lymphocytes were infected with rCDV^RI^Venus(6) at the peak of infection, this was approximately 80% for rCDV^RI^Venus(1)-L_H589Y_. Surprisingly, the percentages of infected Tc-lymphocytes and B-lymphocytes were much lower for rCDV^RI^Venus(1)-L_H589Y_-inoculated animals when compared to rCDV^RI^Venus(6)-inoculated ferrets (approx. 20% vs 50%–60%, respectively). Percentages of infection of DCs and monocytes were similar in both groups; granulocytes were hardly infected by CDV. Moreover, we compared phenotypes at the peak of infection (6 dpi) for rCDV^RI^Venus(6) and rCDV^RI^Venus(1)-L_H589Y_. For rCDV^RI^Venus(6), the majority of infected cells were B-lymphocytes, while for rCDV^RI^Venus(1)-L_H589Y_, similar amounts of T- and B-lymphocytes were infected (Fig. 5C and D).

Consequently, in addition to overall lymphopenia observed (Fig. 4C), we also detected a change in the relative fraction of WBC (Fig. 5E and F). At 4 dpi, a relative decrease in Th- and Tc-lymphocytes was detected for both groups. For rCDV^RI^Venus(6)-inoculated ferrets, all lymphocyte fractions declined further over time, resulting in no T-lymphocytes at 10 dpi and only limited numbers of B-lymphocytes at 13 dpi (*N* = 1 for 10 and 13 dpi) (Fig. 5E). Relative lymphocyte fractions also decreased for rCDV^RI^Venus(1)-L_H589Y_-inoculated ferrets, but at all evaluated time points, a fraction of B-lymphocytes remained (Fig. 5F). At 20 dpi, T-lymphocyte fractions were still low (<10%), but B-lymphocyte, DC, and monocyte fractions recovered, indicating overall rehabilitation of the immune system of rCDV^RI^Venus(1)-L_H589Y_-inoculated ferrets.

### rCDV^RI^Venus(1)-L_H589Y_ in lymphoid tissues

**Fig 6:**
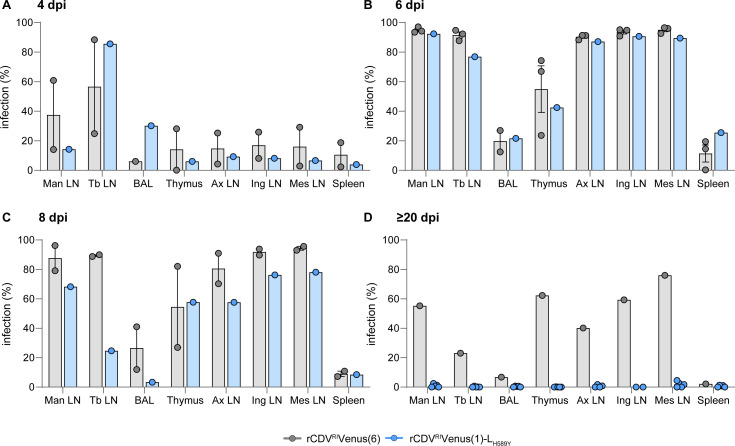
Detection of Venus^+^ cells for rCDV^RI^Venus(6)- and rCDV^RI^Venus(1)-L_H589Y_-infected animals in BAL cells and different lymphoid tissues over time. (**A–D**) Infection percentages were measured in single-cell suspensions from different tissues via flow cytometry at (**A**) 4, (**B**) 6, (**C**) 8, and (**D**) 20–23 dpi. The percentage of Venus^+^ cells within the singlet gate is shown. Symbols represent individual animals, bars the average, and error bars depict the SEM. Man LN, mandibular lymph node; Tb LN, tracheobronchial lymph node; BAL, broncho-alveolar lavage; Ax LN, axillary lymph node; Ing LN, Inguinal lymph node; Mes LN, mesenteric lymph node.

Last, we compared the spread of rCDV^RI^Venus(1)-L_H589Y_ to the spread of rCDV^RI^Venus(6) in lymphoid tissues and lungs over time (Fig. 6). Organs were harvested upon euthanization, and the data presented depict individual animals. At the early time points (4 and 6 dpi), infection percentages of mandibular, tracheobronchial, axillar, inguinal, and mesenteric lymph nodes as well as broncho-alveolar lavage cells, thymus, and spleen were similar for both groups (Fig. 6A and B). At 8 dpi, an overall lower amount of rCDV^RI^Venus(1)-L_H589Y_ infection when compared to rCDV^RI^Venus(6) infection was observed for different evaluated tissues (Fig. 6C). Importantly, this effect was even more pronounced at 20 dpi when rCDV^RI^Venus(1)-L_H589Y_-inoculated ferrets had largely cleared their infection from all lymphoid tissues, while a considerable amount of rCDV^RI^Venus(6)-infected cells could still be detected in all tissues [*N* = 1 for rCDV^RI^Venus(6)] (Fig. 6D). This finding confirms that rCDV^RI^Venus(1)-L_H589Y_-inoculated ferrets had overcome their infection.

## DISCUSSION

We genetically modified and compared five rCDVs to rCDV^RI^Venus(6) (33). Previously, we have reported that rCDV^RI^Venus(6) causes severe distemper in ferrets and raccoons (18, 34). In direct comparison to rCDV^RI^Venus(6), two rCDVs proved to be insufficiently attenuated, while rCDV^RI^Venus(6)-L_EGFP_ was too attenuated. Both polymerase point mutation rCDVs were also attenuated but were still immuno-pathogenic and were shed efficiently. rCDV^RI^Venus(1)-L_H589Y_ appeared to cause more “measles-like” disease with its lower CFR and severity score.

Other rCDV strains were previously reported to be attenuated (10, 19, 22, 25, 28, 35, 36). “Nectin-4-blind” viruses largely recapitulated the immuno-pathogenesis of measles in humans, resulting in lymphocyte infection and depletion. However, these viruses were not able to infect epithelial cells, were not transmitted, and infected animals did not show clinical manifestations (28, 35). These three aspects are crucial to fully recapitulate measles pathogenesis.

In the context of WT-based CDV, the deletion of the C protein (23, 37, 38) did not result in its attenuation. We aimed to delete the C protein by knocking out the start codon and introducing three in-frame stop codons shortly after at positions 2, 6, and 9 of the C protein. Likewise, a similar attenuation strategy of knocking out the start codon and introducing two in-frame stop codons at positions 9 and 12 also did not result in attenuation (39). However, recently it was shown that a truncated CDV-C protein can be expressed from a downstream start codon, which still maintained functionality (36). We assume that the same may have happened in our study. In our study, cloning an ATU at position 1 of the genome or an attenuating point mutation in the L gene led to lethal disease with CNS involvement in some inoculated ferrets. Previously, when EGFP was inserted at position 1 of the genome of highly pathogenic rCDV^5804P^, *n* = 2/3 ferrets survived the infection, and *n* = 1/3 ferrets was sacrificed at the peak of disease signs (22). This difference may be related to the intrinsic virulence of the CDV strains used. Recombinant CDV^5804P^ expressing EGFP at position 6 (non-attenuated virus) infected 40% of T-cells, 57% of B-cells, and 0% of monocyte/macrophage at 7 dpi. In contrast, our comparable rCDV^RI^Venus(6) infected approx. 90% of Th-cells, 50% of Tc-cells, similar percentages of B-cells (60%), and approx. 20% of DCs at 6 dpi, indicating an intrinsic higher virulence compared to rCDV^5804P^.

In contrast, EGFP within the L gene highly attenuated CDV. Although inoculation of ferrets with rCDV^RI^Venus(6)-L_EGFP_ resulted in productive replication and transient lymphopenia, clinical signs were not observed. Moreover, the kinetics of replication largely differed from all other tested viruses, and only low amounts of CDV were shed. This would likely not lead to efficient transmission, an important hallmark of morbilliviruses. Previously, a similar approach was used to attenuate RPV (24). *In vitro*, the attenuated RPV strain and the parental strain grew in B95a cells at the same rate, but *in vivo* attenuation was observed, resulting in mild clinical signs and transient lymphopenia in cattle infected with the attenuated strain. Similarly, modification of CDV^5804P^ in the same way resulted in over-attenuation (25).

In this study, attenuating rCDV by introducing an ATU at position 1 (22) in combination with the point mutation H589Y in L (26) resulted in the currently best available model to mimic WT MeV. With this model, we met the majority of our pre-defined goals to accurately reflect the pathogenesis of measles: (i) rCDV^RI^Venus(1)-L_H589Y_ had an identical tropism to MeV by using CD150 and nectin-4. (ii) All rCDV^RI^Venus(1)-L_H589Y_-inoculated ferrets survived infection, showing that rCDV^RI^Venus(1)-L_H589Y_ infection is self-limiting. (iii) rCDV^RI^Venus(1)-L_H589Y_ infection resulted in transient viremia, and (iv) infected cells spread systemically to all screened lymphoid tissues. We saw similar kinetics when comparing rCDV^RI^Venus(1)-L_H589Y_ to MeV in NHP, although the peak of infection was slightly earlier (6 vs 9 dpi, respectively) (40). Infection of peripheral lymphocytes and lymphoid organs was more extensive in our CDV-ferret model than observed for MeV in NHPs. While during the peak of infection approximately 5%–10% of peripheral lymphocytes are infected by MeV, we observed almost 40% infection with rCDV^RI^Venus(1)-L_H589Y_ (40). Similarly, infection percentages of lymphoid tissues were also higher in ferrets, but rCDV^RI^Venus(1)-L_H589Y_ was nevertheless cleared from lymphoid tissues by the end of the study. (v) As seen for MeV in NHP (40), rCDV^RI^Venus(1)-L_H589Y_ only transiently shed from the upper respiratory tract and (vi) did not result in neurological signs. (vii) rCDV^RI^Venus(1)-L_H589Y_ infection should have caused transient lymphopenia. However, in this model, lymphocyte counts did not fully recover, although systemic re-population of initially depleted myeloid cells and B-lymphocytes could be observed at 21 dpi. Previously, in a different ferret model with rCDV^5804P^ expressing EGFP at position 1, WBC counts had largely recovered by 28 dpi and fully recovered by 35 dpi, a time window that we did not explore in our study (22).

The immunosuppressive nature of rCDV^RI^Venus(1)-L_H589Y_ will allow for extension of the studies of measles-induced immune amnesia outside of the NHP model. Previously, vaccine-acquired immunity to influenza virus was shown to be compromised in ferrets after infection with nectin-4-blind CDV (10). Similarly, the effect of rCDV^RI^Venus(1)-L_H589Y_ on the immune repertoire will give insights into functional impairment, depletion, and conditions for re-population of specific memory lymphocytes. Moreover, we envision potential applications of this model for intervention studies. Although current measles vaccines are safe and effective for many, young infants and immunocompromised patients cannot be vaccinated with those live-attenuated vaccines. This model gives a platform to test new vaccine candidates. Lastly, antivirals interfering with the viral life cycle can also be tested in this model.

## MATERIALS AND METHODS

### Design of rCDV variants

All viruses were recombinant viruses expressing the fluorescent reporter protein Venus from an ATU. The recombinant CDVs were based on the sequence of CDV strain Rhode Island obtained from a naturally infected raccoon (33) with changes predicted to rationally attenuate the viruses. rCDV^RI^Venus(6) and rCDV^RI^Venus(6)-L_EGFP_ have been described previously (33). rCDV^RI^Venus(1), rCDV^RI^Venus(6)-ΔC, rCDV^RI^Venus(6)-L_H589Y_, and rCDV^RI^Venus(1)-L_H589Y_ were recovered from plasmids pCDV^RI^Venus(1), pCDV^RI^Venus(6)-ΔC, pCDV^RI^Venus(6)-L_H589Y_, and pCDV^RI^Venus(1)-L_H589Y_, respectively, as previously described (33). To generate pCDV^RI^Venus(1), pCDV^RI^ (33) was digested with Not I (in the plasmid backbone) and BbvC I (in N), and the intervening fragment was replaced (by Gibson Assembly) with a gBlock (Integrated DNA Technologies, IDT) containing a Venus-encoding transcription unit upstream of the N gene (position 1). To generate pCDV^RI^Venus(6)-ΔC, pCDV^RI^Venus(6) was digested with BbvC I (in N) and Sal I (downstream of the C open reading frame in P), and the intervening fragment was replaced (by Gibson Assembly) with two overlapping PCR-generated amplicons containing nucleotide mutations to knock out the start codon of the C protein and introduce three in-frame stop codons at C codon positions 2, 6, and 9 in a strategy similar to that used previously for MeV (41); the coding sequence of P is not affected by the introduced mutations. To generate pCDV^RI^Venus(6)-L_H589Y_, pCDV^RI^Venus(6) was digested with Swa I (upstream of the modified region in L) and Xho I (downstream of the modified region in L), and the intervening fragment was replaced (by Gibson Assembly) with a gBlock (IDT) containing a nucleotide mutation to convert histidine at position 589 of the L protein to tyrosine; this mutation has been previously shown to attenuate CDV in ferrets (26). To generate pCDV^RI^Venus(1)-L_H589Y_, pCDV^RI^Venus(1) was digested with Aat II (upstream of the modified region in L) and Xho I (downstream of the modified region in L), and the intervening fragment was replaced (by ligation) with the equivalently digested fragment from pCDV^RI^Venus(6)-L_H589Y_. An overview of viral constructs can be found in Fig. 1.

### Cells

Stocks were grown on Vero-dogSLAM cells (VDS; a kind gift from Dr. Y. Yanagi) (42) or on canine lymphoblastoid cell line (CLBL) (43), and the viral load was determined by endpoint titration on VDS. To determine receptor usage Vero cells, VDS and Vero-dogNectin-4 (VdN4, a kind gift from Dr. C. Richardson) cells were used. Vero, VDS, and VdN4 cells were grown in Dulbecco’s modified Eagle medium, and CLBL was grown in RPMI-1640, supplemented with 10% fetal bovine serum (vol/vol), penicillin (100 IU/mL), streptomycin (100 µg/mL), and L-glutamine (2 mM).

### Animal study design

Ferrets (*Mustela putorius furo*) were intratracheally inoculated with 1 × 10^4^ TCID_50_ of rCDVs. Only male ferrets were used in this study to allow for more frequent blood sampling because of their larger body size. Animals inoculated with rCDV^RI^Venus(6) were previously described, and the animals that reached their humane endpoints were used as a control group (34). The course of infection was monitored for up to 23 days post-inoculation (dpi). Throat, nose, and rectal swabs as well as WBC from whole blood and tissues were collected at various time points. We recorded the core body temperature through a temperature logger (StarOddi DST micro-T), which was implanted intra-peritoneally on average 2 weeks before the start of the study. Ferrets were euthanized at 2, 4, 6, 8, 10, 13, 14, 15, 16, 17, 20, 21, and 23 dpi by exsanguination under ketamine-medetomidine anesthesia. We retrospectively calculated the severity score of each animal and compared those to ferrets inoculated with MeV which does not replicate in ferrets and can therefore be considered negative controls (34). The severity score forms an average score of body temperature, body weight, lymphocyte counts, virus isolation from throat swabs, and infection of lymphocytes. Each parameter is classified from 0 to 3 based on severity (0 being the lowest and 3 the highest severity), and the average score of all parameters per animal is calculated (Table S1). Macroscopic fluorescence was detected using a custom-made lamp containing six 5-Volt LEDs (Luxeon Lumileds, Lambertian, cyan, peak emission 490–495 nm) mounted with D480/40 bandpass filters (Chroma), and photographs were taken using a Nikon D80 SLR camera mounted with a Dark Reader camera filter (Clare Chemical Research) (6). All numbers of animals included in different analyses are summarized in Table S2.

### Flow cytometry

Frequencies of infected cells and WBC subsets in blood and tissues were determined by flow cytometry. WBCs were isolated from whole blood after red blood cell lysis with buffer (Roche). At necropsy, lymphoid tissues were collected in phosphate-buffered saline (PBS) for preparation of single-cell suspensions, processed by cutting into smaller pieces, and further dissociated with gentleMACS (Miltenyi Biotec). Single-cell suspensions from the tissues were then passed through a 100-µm filter and isolated in PBS. For WBC phenotyping, cells were stained with CD4^APC^ (Clone MM02; 1:25), CD8^PE-Cy7^ (Clone: OKT3; 1:25), CD11b^AmCyan^ (Clone: M1/70, 1:25), and HLA-DR^APC-Cy7^ (Clone: L243; 1:25) antibodies; on average, more than 4 × 10^5^ events were recorded. Subsets were defined using FSC and SSC as well as the cell-surface markers. Th-lymphocytes were defined as FSC^low^, CD11b^−^, HLA-DR^−^, CD4^+^; Tc-lymphocytes were defined as FSC^low^, CD11b^−^, HLA-DR^−^, CD8^+^; B-lymphocytes were defined as FSC^low^, CD11b^−^, HLA-DR^+^; DCs were defined as FSC^high^, CD11b^high^, HLA-DR^high^; monocytes were defined as FSC^low^, CD11b^high^, HLA-DR^inter^; granulocytes were defined as FSC^low^, CD11b^high^, HLA-DR^−^. The percentage of infected cells was determined by detection of Venus^+^ cells in each subset. All data were analyzed using FlowJo software. A gating strategy can be found in Fig. S3.

### Virus isolation

Throat, nose, and rectal swabs were resuspended in a 2-mL virus transport medium (VTM). Upon one freeze-thaw cycle, virus-containing VTM was titrated on VDS in a threefold dilution series. For virus isolation from WBC, 80,000 WBCs were diluted in 1 mL, and then a twofold dilution series was performed on VDS. Four days post virus isolation, viral load was determined by fluorescence detection, and virus titer (TCID_50_/mL) was calculated using the Reed-Muench method (44).

## SUPPLEMENTAL MATERIAL

Supplemental material jvi.01850-23-s0001.pdf Tables S1 and S2; Figures S1 to S3.

ASM does not own the copyrights to Supplemental Material that may be linked to, or accessed through, an article. The authors have granted ASM a non-exclusive, world-wide license to publish the Supplemental Material files. Please contact the corresponding author directly for reuse.
