# Supplementary material for: Rational attenuation of canine distemper virus (CDV) to develop a morbillivirus animal model that mimics measles in humans
Source: J Virol. 2024 Feb 28;98(3):e01850-23. doi: 10.1128/jvi.01850-23 (PMC10949419; doi:10.1128/jvi.01850-23)
Supplement: Supplemental material — Tables S1 and S2; Figures S1 to S3. [file jvi.01850-23-s0001.pdf]

**Supplemental Table 1:** Severity scores

| | **0** | **1** | **2** | **3** |
|---|---|---|---|---|
| **Body temperature** (°C, relative to 0 dpi) | <0.25 | 0.25 - <0.75 | 0.75 - <1.25 | ≥1.25 |
| **Body weight** (% difference to 0 dpi) | >97.5 | 95-97.5 | 90-95 | <90 |
| **Lymphocyte counts** (% difference to 0 dpi) | >80 | 60-80 | 30-60 | <30 |
| **Virus isolation from throat swab** ($TCID_{50}$/ml) | <1 | 1 – 10 | 10 – 1,000 | >1,000 |
| **Infection of lymphocytes** (%) | <0.1 | 0.1 – 5 | 5 – 15 | > 15 |

**Supplemental Table 2:** Animals included in different analyses. The animals infected with rCDV$^{RI}$Venus(6) were previously described (Laksono et al. mSphere 2023).

| Virus | Animal | Day of Euthanasia | Analyses | | | | | | | |
|---|---|---|---|---|---|---|---|---|---|---|
| | | | Body temp | Lymphocyte count | CFR | Shedding | Viremia (FACS) | Viremia (isolation) | WBC phenotyping | Infection of lymphoid tissues |
| rCDV$^{RI}$Venus(6) | 01 | 2 | X | X | | X | X | X | X | |
| | 02 | 4 | X | X | | X | X | X | X | X |
| | 03 | 4 | X | X | | X | X | X | X | X |
| | 04 | 6 | X | X | | X | X | X | X | X |
| | 05 | 6 | X | X | | X | X | X | X | X |
| | 06 | 6 | X | X | | X | X | X | X | X |
| | 07 | 8 | X | X | | X | X | X | X | X |
| | 08 | 8 | X | X | | X | X | X | X | X |
| | 09 | 10 | X | X | | X | X | X | X | |
| | 10 | 13 | X | X | X | X | X | X | X | |
| | 11 | 13 | X | X | X | X | X | X | X | |
| | 12 | 14 | X | X | X | X | X | X | (X), day 6 | |
| | 16 | 15 | X | X | X | X | X | X | X | |
| | 17 | 15 | | X | X | X | X | X | X | |
| | 18 | 16 | X | X | X | X | X | X | (X), day 6 | |
| | 19 | 17 | X | X | X | X | X | X | (X), day 6 | |
| | 20 | 17 | | X | X | X | X | X | X | |
| | 21 | 20 | | X | X | X | X | X | X | X |
| | 23 | 23 | | | X | | | | | |
| | 24 | 23 | | | X | | | | | |
| rCDV$^{RI}$Venus (1) | 25 | 4 | X | X | | X | X | X | | |
| | 26 | 6 | X | X | | X | X | X | | |
| | 27 | 8 | X | X | | X | X | X | | |
| | 28 | 13 | X | X | X | X | X | X | | |
| | 29 | 20 | X | X | X | X | X | X | | |
| rCDV$^{RI}$ Venus (6)-ΔC | 37 | 13 | X | X | X | X | X | X | | |
| | 38 | 13 | X | X | X | X | X | X | | |
| | 39 | 13 | X | X | X | X | X | X | | |

| Virus | Animal | Day of Euthanasia | Body temp | Lymphocyte count | CFR | Shedding | Viremia (FACS) | Viremia (isolation) | WBC phenotyping | Infection of lymphoid tissues |
|---|---|---|---|---|---|---|---|---|---|---|
| rCDV$^{RI}$Venus(6)-L$_{EGFP}$ | 40 | 6 | X | X | | X | X | X | | |
| | 41 | 8 | X | X | | X | X | X | | |
| | 42 | 10 | X | X | | X | X | X | | |
| | 44 | 23 | X | X | X | X | X | X | | |
| | 45 | 23 | X | X | X | X | X | X | | |
| | 82 | 21 | X | X | X | X | X | X | | |
| | 83 | 21 | X | X | X | X | X | X | | |
| | 84 | 21 | X | X | X | X | X | X | | |
| rCDV$^{RI}$Venus(6)-L$_{H589Y}$ | 47 | 8 | X | X | | X | X | X | | |
| | 49 | 14 | X | X | X | X | X | X | | |
| | 50 | 23 | X | X | X | X | X | X | | |
| | 51 | 23 | X | X | X | X | X | X | | |
| | 79 | 20 | X | X | X | X | X | X | | |
| | 80 | 20 | X | X | X | X | X | X | | |
| | 81 | 20 | X | X | X | X | X | X | | |
| rCDV$^{RI}$Venus(1)-L$_{H589Y}$ | 52 | 4 | X | X | | X | X | X | X | X |
| | 53 | 6 | X | X | | X | X | X | X | X |
| | 54 | 8 | X | X | | X | X | X | X | X |
| | 55 | 23 | X | X | X | X | X | X | X | X |
| | 57 | 23 | X | X | X | X | X | X | X | X |
| | 76 | 20 | X | X | X | X | X | X | X | X |
| | 77 | 20 | X | X | X | X | X | X | X | X |
| | 78 | 20 | X | X | X | X | X | X | X | X |
| | 85 | 21 | X | X | X | X | X | | | |
| | 86 | 21 | X | X | X | X | X | | | |
| | 87 | 21 | X | X | X | X | X | | | |

Grey area: not intended for analysis; no X: data analysis/recording failed; X: included into analysis. Animals that were euthanized up to or on day 10 or on or after day 20 (exemption animal 21) were euthanized as per protocol schedule. All animals euthanized in between (day 13-17) reached humane endpoints. As exemption, animal 21 was scheduled for euthanasia on day 23 but reached its humane endpoint on day 20. WBC phenotyping varies per time point evaluated as not all samples were stained at every time point. For a specific number of analyzed samples per time point (n) refer to Figure 5 in the main manuscript. For rCDV$^{RI}$Venus(6)-inoculated animals, animals 23 and 24 were only included for case fatality rate (CFR) calculations as both survived the infection; all other analysis was exclusively performed on animals that reached their humane endpoints or were sacrificed at early time points.

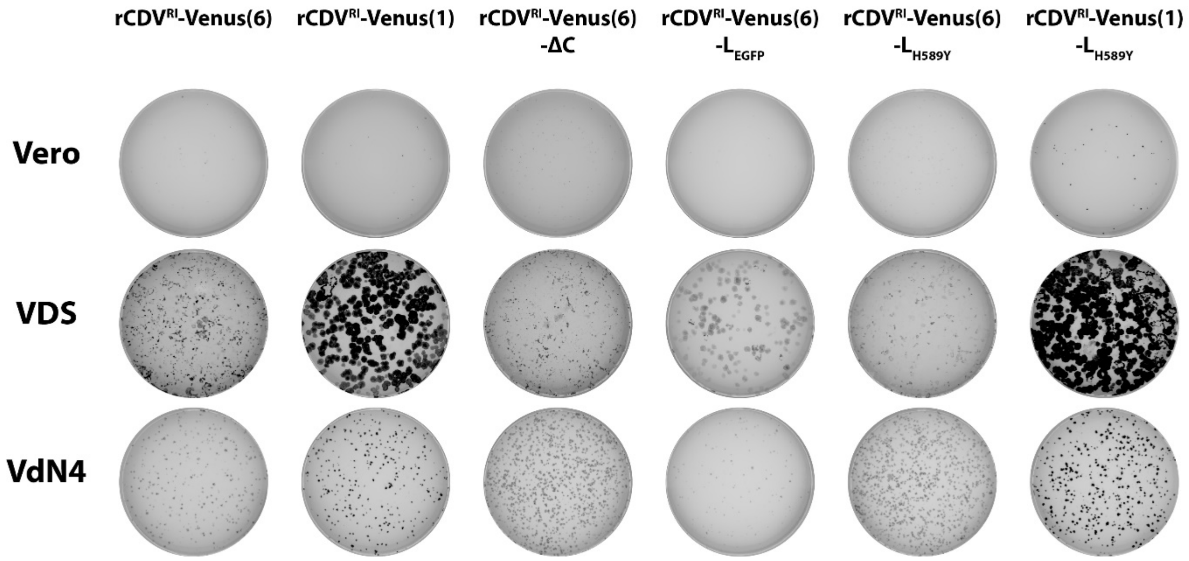


**Supplemental Figure 1. rCDVs use CD150 and nectin-4 as cellular receptors.** Infection of Vero cells, Vero cells overexpressing dog-CD150 (VDS) or dog-nectin-4 (VdN4) with different rCDVs resulted in productive infection of the latter two. Infected cells express the fluorescent protein Venus which is shown in grey/black.

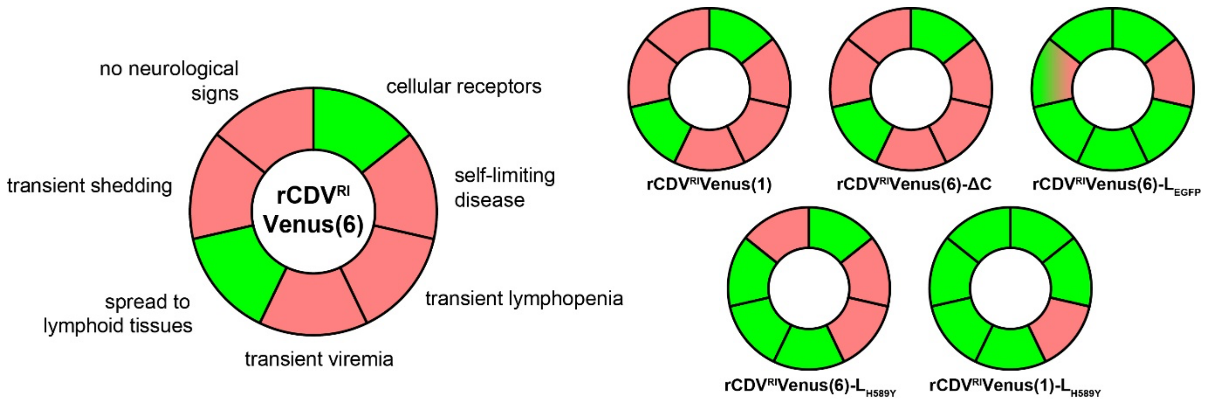


**Supplemental Figure 2. Measles-like characteristics observed in rCDV-infected ferrets.** All rCDVs were evaluated for the use of cellular receptors CD150 and nectin-4, disease severity and CFR (self-limiting disease), lymphopenia, viremia, spread to lymphoid tissues, shedding and lack of neurological signs. Red indicates that a criterium is not met, green indicates that a criterium is met. $rCDV^{RI}Venus(6)\text{-}L_{EGFP}$ appeared to be too attenuated as no clinical disease could be observed (therefore indicated in red) and the virus could only be isolated for a short time and to low titers (therefore indicated in red-green).

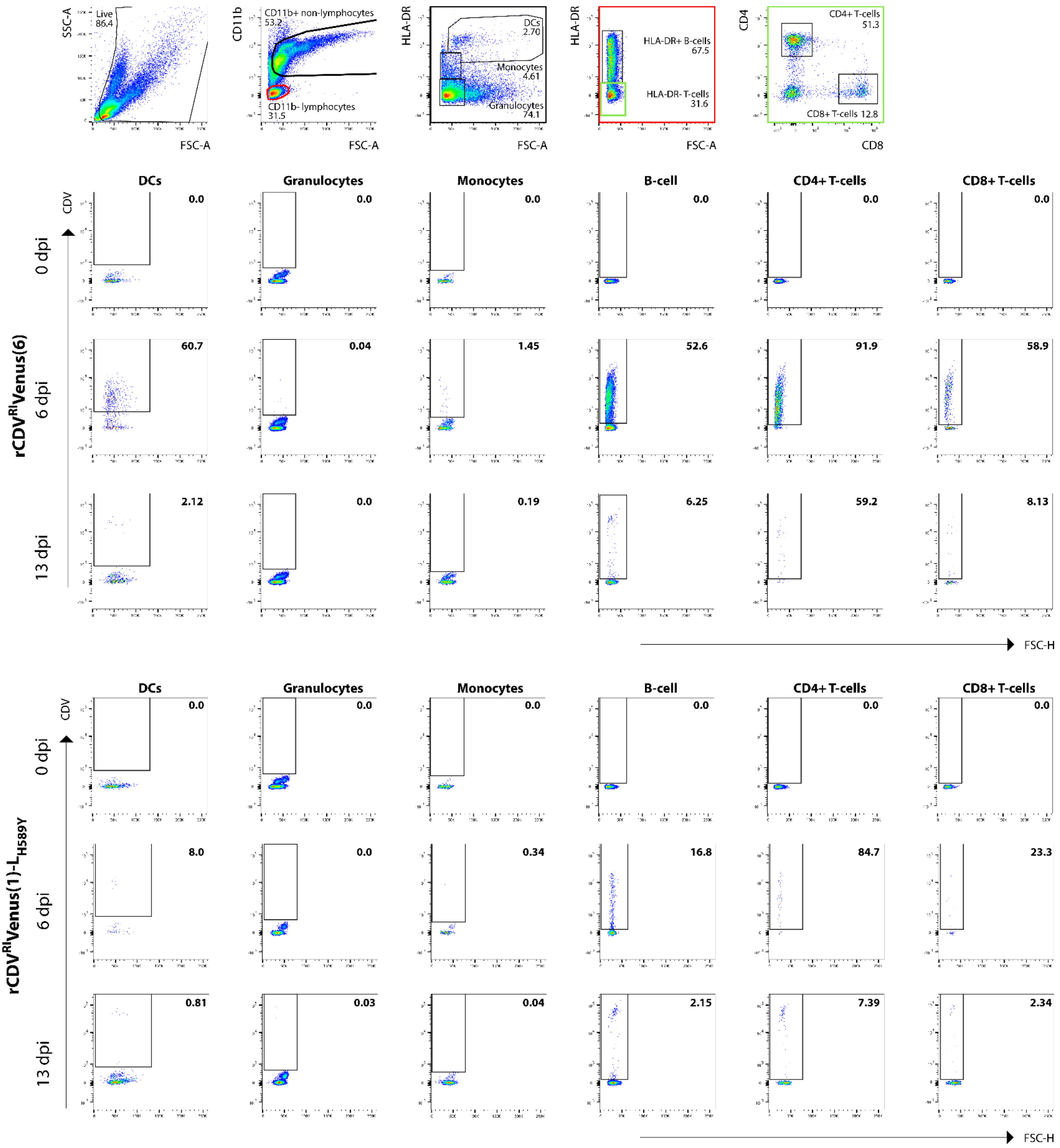


**Supplemental Figure 3: gating strategy**. White blood cells were isolated, stained and characterized by flow cytometry based on FSC, SSC and cell surface markers. Per sub-population Venus-positive cells as a measure of infection were detected. Top row indicates gating strategy to define cell subsets, middle three rows show infection percentages for one ferret infected with rCDV$^{RI}$Venus(6) 0, 6 and 13 dpi and bottom three rows show infection percentages for one ferret infected with rCDV$^{RI}$Venus(1)-L$_{H589Y}$ at the same time points.
